# Artery Tertiary Lymphoid Organs: Powerhouses of Atherosclerosis Immunity

**DOI:** 10.3389/fimmu.2016.00387

**Published:** 2016-10-10

**Authors:** Changjun Yin, Sarajo Kumar Mohanta, Prasad Srikakulapu, Christian Weber, Andreas J. R. Habenicht

**Affiliations:** ^1^Institute for Cardiovascular Prevention, Ludwig-Maximilians-University, Munich, Germany; ^2^German Centre for Cardiovascular Research (DZHK), Partner Site Munich Heart Alliance, Munich, Germany; ^3^Cardiovascular Research Center (CVRC), University of Virginia, Charlottesville, VA, USA

**Keywords:** atherosclerosis, aging, adventitia, autoimmune responses, artery tertiary lymphoid organs

## Abstract

Artery tertiary lymphoid organs (ATLOs) are atherosclerosis-associated lymphoid aggregates with varying degrees of complexity ranging from small T/B-cell clusters to well-structured lymph node-like though unencapsulated lymphoid tissues. ATLOs arise in the connective tissue that surrounds diseased arteries, i.e., the adventitia. ATLOs have been identified in aged atherosclerosis-prone hyperlipidemic apolipoprotein E-deficient (ApoE^−/−^) mice: they are organized into distinct immune cell compartments, including separate T-cell areas, activated B-cell follicles, and plasma cell niches. Analyses of ATLO immune cell subsets indicate antigen-specific T- and B-cell immune reactions within the atherosclerotic arterial wall adventitia. Moreover, ATLOs harbor innate immune cells, including a large component of inflammatory macrophages, B-1 cells, and an aberrant set of antigen-presenting cells. There is marked neoangiogenesis, irregular lymphangiogenesis, neoformation of high endothelial venules, and *de novo* synthesis of lymph node-like conduits. Molecular mechanisms of ATLO formation remain to be identified though media vascular smooth muscle cells may adopt features of lymphoid tissue organizer-like cells by expressing lymphorganogenic chemokines, i.e., CXCL13 and CCL21. Although these data are consistent with the view that ATLOs participate in primary T- and B-cell responses against elusive atherosclerosis-specific autoantigens, their specific protective or disease-promoting roles remain to be identified. In this review, we discuss what is currently known about ATLOs and their potential impact on atherosclerosis and make attempts to define challenges ahead.

## Atherosclerosis

Atherosclerosis leading to cardiovascular diseases is the major cause of death worldwide (1–8). The pathological hallmark of atherosclerosis is the atherosclerotic plaque in the intima layer of the arterial wall. Plaques ultimately clog the artery with life-threatening consequences, resulting in tissue infarcts such as myocardial infarcts (heart attacks) and stroke (9). Little is known about molecular mechanisms of atherosclerosis. Health organizations have proposed recommendations regarding risk factor reduction (smoking, diabetes mellitus, obesity, hypertension, hyperlipidemia, and sedentary lifestyle). The most important, but least understood, risk factor is aging. However, there is currently no clue as to the mechanisms of its impact on atherosclerosis progression. A generally accepted hypothesis is that the immune system is critically involved in the pathogenesis of atherosclerosis, but major issues of atherosclerosis immunity remain to be addressed: it is not clear where and when atherosclerosis-specific immune responses are organized; what are the relative contributions of the innate *vis-à-vis* the adaptive immune systems during the various stages of the disease; and, most importantly, is atherosclerosis a *bona fide* antigen-dependent autoimmune disease or a chronic autoinflammatory condition? Answers to these questions are needed to develop therapeutic strategies to directly target the atherosclerotic plaque in the intima of arteries.

## Immune Hypothesis of Atherosclerosis

Each innate and adaptive immune cell lineage and their subtypes has been implicated in the pathogenesis of atherosclerosis including platelets, neutrophils, monocytes/macrophages, mast cells, various dendritic cell (DC) subsets, numerous T- and B-cell subtypes, and innate lymphoid cells (3, 4, 7, 10–22). However, there is no generally accepted concept which immune cells trigger the disease, at which step distinct subsets promote or attenuate the disease, and how plaque growth unfolds at the molecular level. Indeed, widely different hypotheses have been proposed [reviewed in Ref. (23)].

Concepts regarding atherogenesis have been deduced from observations in mouse models including low-density lipoprotein receptor-deficient (LDLR^−/−^) or apolipoprotein E-deficient (ApoE^−/−^) mice (24) and human tissue specimens. Mouse models on hyperlipidemic backgrounds have been generated to disrupt one or more molecules that control the systemic immune system. The worrying fact of the matter, however, is that – given the complex nature of the disease involving multiple genetic and life-style- and aging-driven risk factors – atherosclerosis research is in a dismal state.

Fundamental questions remain: the specific roles of each immune cell subset and their interplay, the timing and sites of their actions, the relative shares of the innate and adaptive immune systems in the organization of atherosclerosis immune responses over time, and the impacts and location of disease-causing and disease-suppressing leukocyte subsets, all remain to be determined. The major challenge, however, concerns the principal nature of the underlying disease-causing immune responses: Is plaque formation a chronic autoinflammatory tissue reaction (without generation of autoimmune B- or T-cells) or are elusive disease-causing autoantigens driving generation and action of autoimmune lymphocyte subsets?

Thus, atherosclerosis research shares major unanswered questions with other clinically important chronic inflammatory diseases such as rheumatoid arthritis, multiple sclerosis, and inflammatory bowel diseases (25–28). Based on circumstantial evidence, some of these diseases are considered *bona fide* autoimmune diseases although – similar to atherosclerosis – their *disease-triggering autoantigens* have not been identified [see review in Ref. (23, 29, 30)]. Moreover, atherosclerosis-specific immune responses have long been assumed to be organized in atherosclerotic plaques in the intima layer of arteries or systemically in secondary lymphoid organs (SLOs), but the evidence for these views is scarce if not non-existing. Thus, it is safe to say that neither the existence, their nature (T- versus B-cell responses), nor the location of autoimmune reactions in atherosclerosis have been identified.

## Atherosclerotic Plaques

The normal intima layer consists of an endothelial cell monolayer attached to the internal basement membrane (7). Vascular DCs have been described in the intima layer of normal mouse arteries, but their role in the maintenance of artery homeostasis or their impact on disease has not been determined (31, 32). The disease ultimately affects all layers of the arterial wall including the media layer [largely consisting of vascular smooth muscle cells (VSMCs)] and the adventitial layer (the outer connective tissue coat; see below): advanced atherosclerosis can therefore be viewed as a chronic *transmural inflammatory arterial wall disease*. Early plaques undergo major alterations of cellular composition over time: during the early stages of the disease, subsequent or concomitantly with the influx of monocytes into the arterial wall, T-cells home into the intima. VSMCs may migrate into the intima and increase in number by self-renewal. Monocytes/macrophages, T-cells, and VSMCs undergo activation and proliferation during the various stages of the disease. It should be pointed out that other views on the early stages of atherosclerosis emphasize the role of platelets and/or neutrophils (12, 21).

## Staging of Atherosclerosis and Distinct Immune Cell Subsets in Arterial Wall Layers

**Figure 1:**
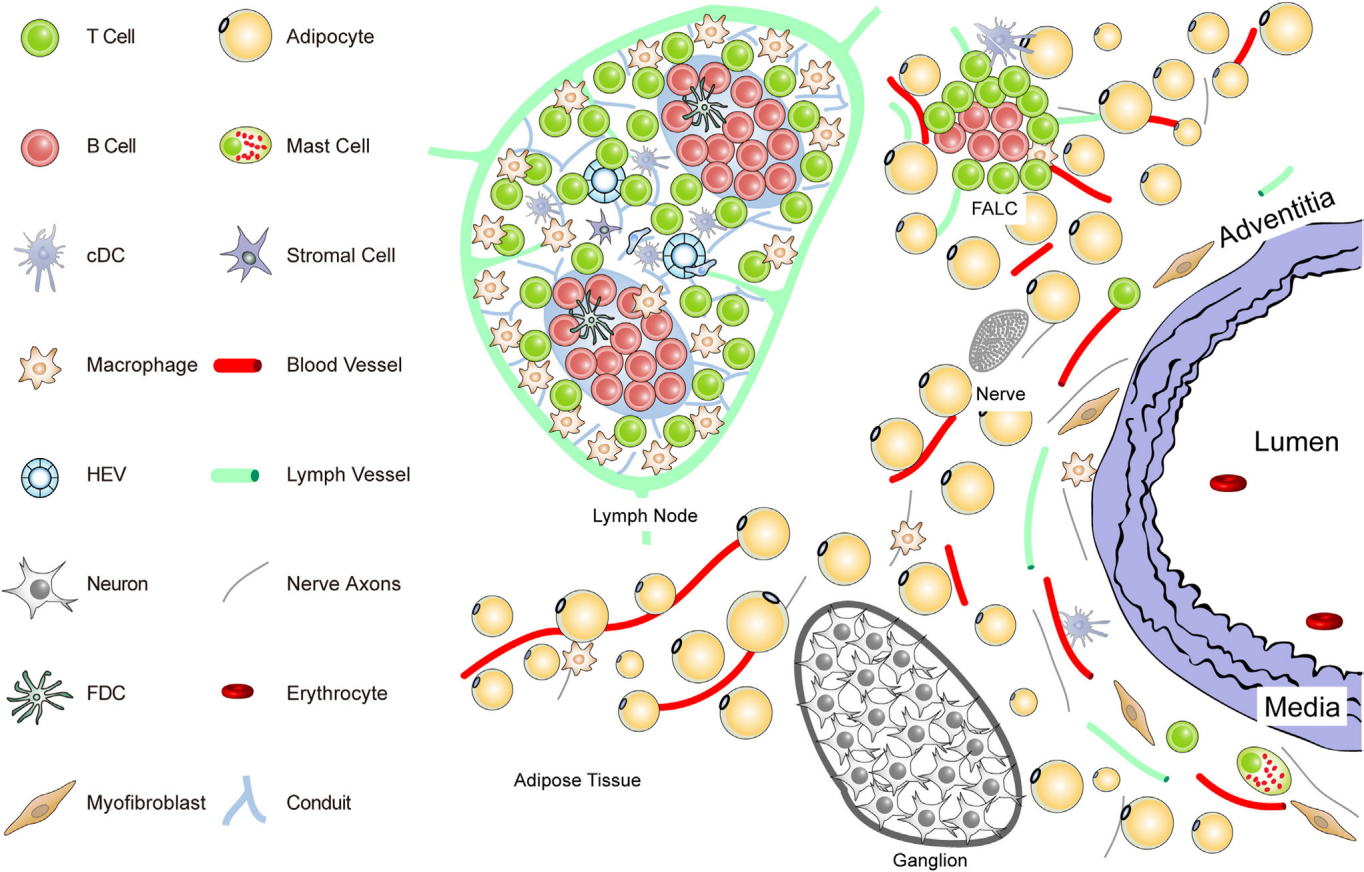
**Anatomy of the normal aorta adventitia and adjacent tissues**. The normal adventitial layer of the mouse aorta constitutively contains T-cells (but no B-cells), tissue macrophages, cDCs, stromal cells or myofibroblasts, and mast cells. The adjacent tissues including the periaortic adipose tissue harbors fat-associated lymphoid clusters (FALCs) in wild-type mice. FALCs represent T/B-cell aggregates of various sizes that are exclusively observed in adipose tissue. In the adventitia, axons of both the sensory and the sympathetic nervous systems have been identified as well as vasa vasorum and lymph vessels. Ganglia of the peripheral nervous system are embedded in the adipose tissue, and their axons reach the external lamina that separates the adventitia from the media layer.

**Figure 2:**
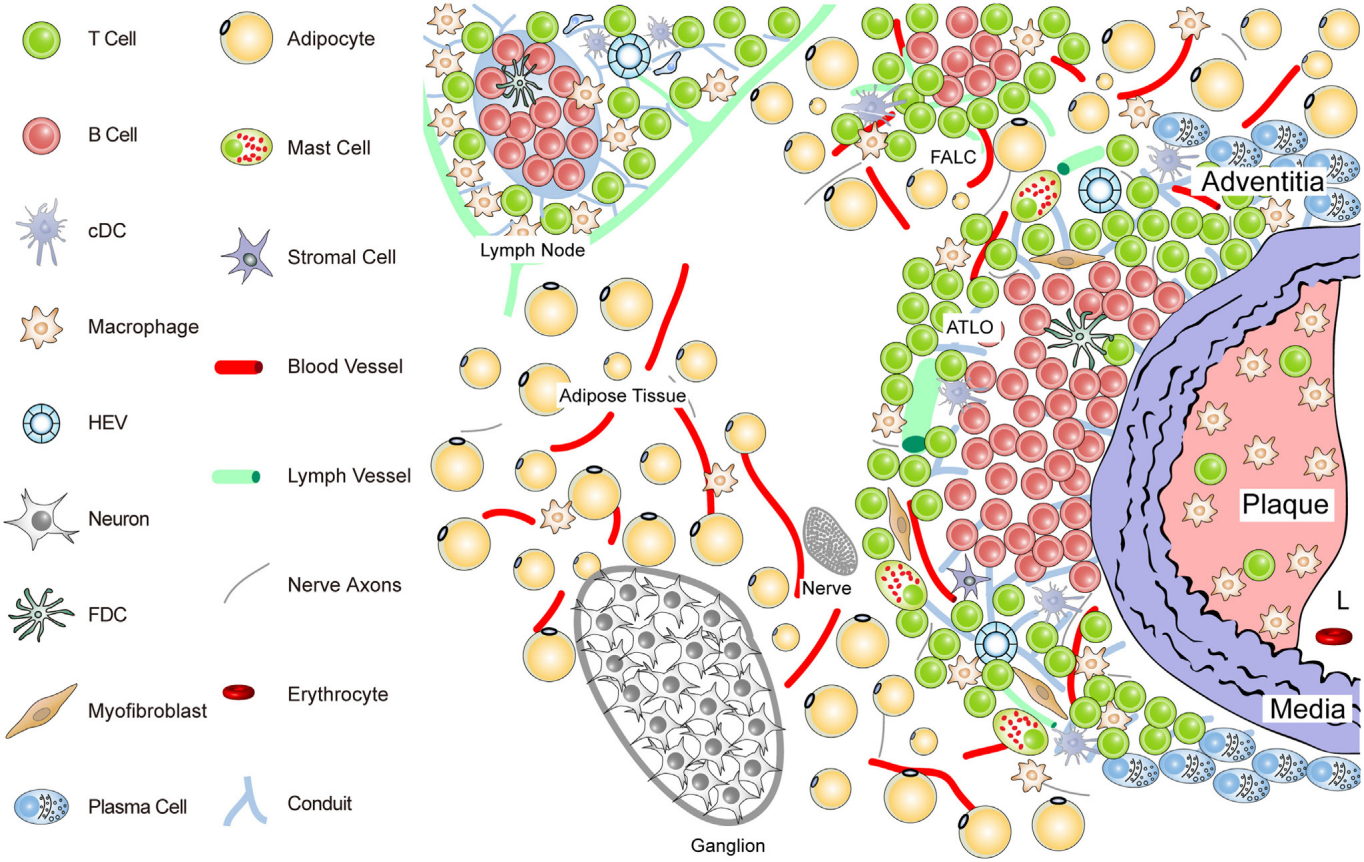
**Anatomy of the diseased aorta adventitia in aged hyperlipidemic mice**. Aged hyperlipidemic mice develop ATLOs in the adventitia layer of the aorta selectively adjacent to atherosclerotic plaques with a preference for the abdominal aorta segment. ATLOs range from small T/B-cell aggregates to well-structured TLOs with separate T- and B-cell areas. The most advanced forms of ATLOs contain B-cell follicles including FDCs, newly formed conduits, HEVs, and aberrant lymph vessels (see Figure 4).

Human atherosclerotic plaques have been staged into types I–VI according to the histological and clinical criteria (9). However, only the late V and VI stages become clinically significant when artery lumen narrowing compromises oxygen supply to the downstream tissue and the plaque becomes unstable. Atherosclerotic plaques in ApoE^−/−^ mice recapitulate some – but not all – features of the different stages of human plaques. Of note, plaques lack B-cells and consequently B-cell follicles including follicular dendritic cells (FDCs). Plaques are also devoid of nerve axons, lymph vessels, high endothelial venules (HEVs), and lymph node-like conduits, which are all critically involved in efficient recruitment of lymphocytes and the subsequent organization of adaptive immune responses (see below) (Figures 1 and 2). It is therefore difficult to envision that immune cells in atherosclerotic plaques participate in adaptive cellular and humoral immune responses though they may be involved in antigen presentation to T-cells through vascular DCs or monocyte-derived DCs (mDCs) that emigrate from plaques to home to SLOs [see contribution of antigen presentation by Hughes et al. (under review)^1^; this Research Topic].

## The Adventitia: An Elusive Connective Tissue Coat of Arteries

A commonly accepted notion states that the adventitia (33–37) forms the *outer connective tissue coat that surrounds blood vessels or arteries*. Given the complexity of the connective tissue surrounding arteries and the lack of size definition together with recent progress in understanding the potential impact of the adventitia in arterial wall remodeling and atherosclerosis, the shortcomings of this definition are apparent (35). However, as we discuss below, the adventitia is a highly active and complex tissue whose roles in the maintenance and homeostasis of the artery is only beginning to be unraveled (38–50).

To delineate the impact the adventitia may have on arterial wall remodeling and atherosclerosis in future studies, a more sophisticated definition should be attempted. The normal connective tissue coat surrounding arteries (in the mouse within 100 μm extending from the aorta external lamina separating the media from the adventitia) includes blood vessels (vasa vasorum), small lymph vessels, and an axon network of both the sympathetic and the sensory peripheral nervous systems (Figure 1).

Unlike the resistance arteries (muscular arterioles) involved in blood pressure regulation, axons of both of these peripheral autonomous nervous systems, but not of the parasympathetic nervous system, reach out to the external lamina; however, they do not cross into the media of medium- and large-sized arteries in mice. Major cellular constituents in the normal adventitia are mast cells, T-cells, and tissue macrophages, a meshwork of fibroblasts or myofibroblasts, classical DCs (cDCs), and most likely pericytes [see contribution of Kranich and Krautler (51); this Research Topic]. Furthermore, the adjacent adipose tissue (containing FALCs) needs to be considered as a tissue in the immediate vicinity of the adventitia (Figure 1). Thus, in mice, the immediate connective tissue coat within a diameter of 100 μm contains myofibroblasts, a loose network of extracellular matrix components, small blood and lymph vessels, and nerve axons. In addition, few tissue macrophages, T-cells, cDCs, and mast cells are important cellular constituents of the normal adventitia (33, 52) (Figure 1).

Each of these immune cell subsets may participate in immune surveillance of arteries (35, 53). Adventitia progenitor cells maintain the endothelial and VSMC pools (52). The vasa vasorum is a network of small blood vessels that reach the external lamina providing nutrients and oxygen to large- and medium-sized arteries (36, 38, 54, 55). Though little is known about their functional impact, conceivably, each of these adventitia constituents could affect arterial wall homeostasis. We have begun to systematically delineate the aorta adventitia of wild-type (WT) mice and hyperlipidemic ApoE^−/−^ mice (Figures 1 and 2). The normal adventitia is neighboring the para-aortic adipose tissue, which contains FALCs recently described in the omentum of the peritoneum of mice [(56); observations; contribution of Caamano et al. (under review)^2^; this Research Topic]. Although little is known about them in atherosclerosis, it is conceivable that FALCs may participate in atherosclerosis immune responses. It is important to distinguish each of the adventitia and adjacent tissues as it is recognized that cells in the adventitia and the adjacent tissues are capable of sensing and responding to a wide range of stimuli (37).

## Discovery and Staging of ATLOs

Tissues that are invaded by antigen are targeted by leukocytes which form – within hours – an acute form of inflammatory infiltrate. This rapid and vigorous action of the innate immune system is carried out with the aim to eliminate the antigen before it can spread and/or inflict harm. However, if the immune system fails to eliminate the invaders, the immune system doubles down on its efforts: the nature of the inflammatory infiltrate will change over time including a reduction of neutrophils, further recruitment of blood monocytes, *de novo* recruitment of T-cells and DCs and form – within days – a predominantly monocyte/macrophage/T-cell/DC-driven inflammatory tissue response (57–59).

Can this type of immune cell infiltrate qualify as a TLO? It probably does or should not qualify for the following reasons: lymphorganogenesis during ontogeny and in adult organisms requires action of lymphorganogenic chemokines, i.e., CCL21 and CXCL13 (60, 61), which are essential for the attraction of B-cells and the formation of T/B-cell aggregates (various contributions in this Research Topic). Without lymphorganogenic chemokines, the immune system is severely impaired (60, 62). There may be exceptions to this paradigm as recent studies on colitis models in mice suggest that the nervous system is not only triggering the earliest forms of lymph node anlagen (63) but also TLO neogenesis in the gastrointestinal tract (64). In addition, early and chronic inflammatory infiltrates without major structural components of SLOs including HEVs, lymph vessels, and conduits, and T-cell areas and B-cell follicles may be less efficient in recruiting and activating naïve lymphocytes to generate T- and B-memory cells in response to antigen [(65); see Ruddle (under review)^3^; this Research Topic].

In 2004, we reported that the number of inflammatory leukocytes, in particular monocyte/macrophages and T-cells, when determined by morphometry of the innominate artery and throughout the arterial tree, increase progressively in the adventitia during aging (66). We systematically studied the relation between plaque and adventitial leukocytes in ApoE^−/−^ mice that were adolescent/young (16 weeks; small atherosclerotic plaques), adult (32 weeks; significant atherosclerosis in the aortic arch; little atherosclerosis in the abdominal aorta); advanced adult (52 weeks; significant aortic arch atherosclerosis; beginning atherosclerosis of the abdominal aorta), and aged (78 weeks; significant atherosclerosis throughout the aorta including abdominal segments) (46).

The results of these studies can be summarized as follows: the inflammatory infiltrate of atherosclerotic plaques decreased in cellularity over time; by contrast, adventitial T-cells and macrophages increased over time in adventitia segments areas that were afflicted with atherosclerosis throughout all major arteries; the size of the adventitial infiltrates correlated with atherosclerotic plaque burden in a highly territorialized way; the ratio between adventitia T-cell density over plaque T-cell density reached a dramatic approximately 80-fold in aged ApoE^−/−^ mice in some segments (46); T-cells were both CD4^+^ and CD8^+^ T helper cells and also included a large T regulatory cell component (see below); the cellularity of the ubiquitous adventitial infiltrate in areas such as the innominate artery and the thoracic aorta could be distinguished from that in the upper abdominal portion of the aorta, however, which was being infiltrated by B-cells at around 52 weeks of age. We therefore suggested that the lymphocyte aggregate of the earliest TLO stage (Stage I) should be characterized by a combined T-/B-cell aggregate. Stage I TLOs have not yet been fully separated T-cell and B-cell compartments, but they are precursors of the well-developed later artery tertiary lymphoid organ (ATLO) stages as indicated by kinetic experiments (41, 46). We suggested three stages of ATLOs.

*ATLO Stage I*: B-cells begin to infiltrate the adventitia as loosely arranged leukocytes without forming distinct T- and B-cell areas; ATLO Stage I emerges parallel to the formation of atherosclerotic plaques in the intima during aging and has not been observed in young mice; this first ATLO stage strongly indicates the action of lymphorganogenic chemokines, i.e., CCL21 and CXCL13, which may be important chemoattractants for T- and B-cells, respectively, and thus ATLO neogenesis. It is noteworthy that not all adventitial leukocyte infiltrates adjacent to atherosclerotic plaques develop into ATLOs. In hyperlipidemic mice, ATLO Stage I, as all subsequent stages, has a strong preference for the upper abdominal aorta adventitia. However, we have little cues as to the molecular mechanisms for this predilection site. *ATLO Stage II*: separate T- and B-cell areas emerge; lymph vessel neogenesis becomes prominent together with a dense network of lymph node-like conduits connecting the arterial wall with newly formed HEVs. *ATLO Stage III*: a well-structured ATLO contains separate T-cell and B-cell follicles with activated germinal centers (GCs) including FDCs, plasma cell (PC) niches, prominent lymph vessel, and blood vessel neogenesis of vasa vasorum.

**Figure 3:**
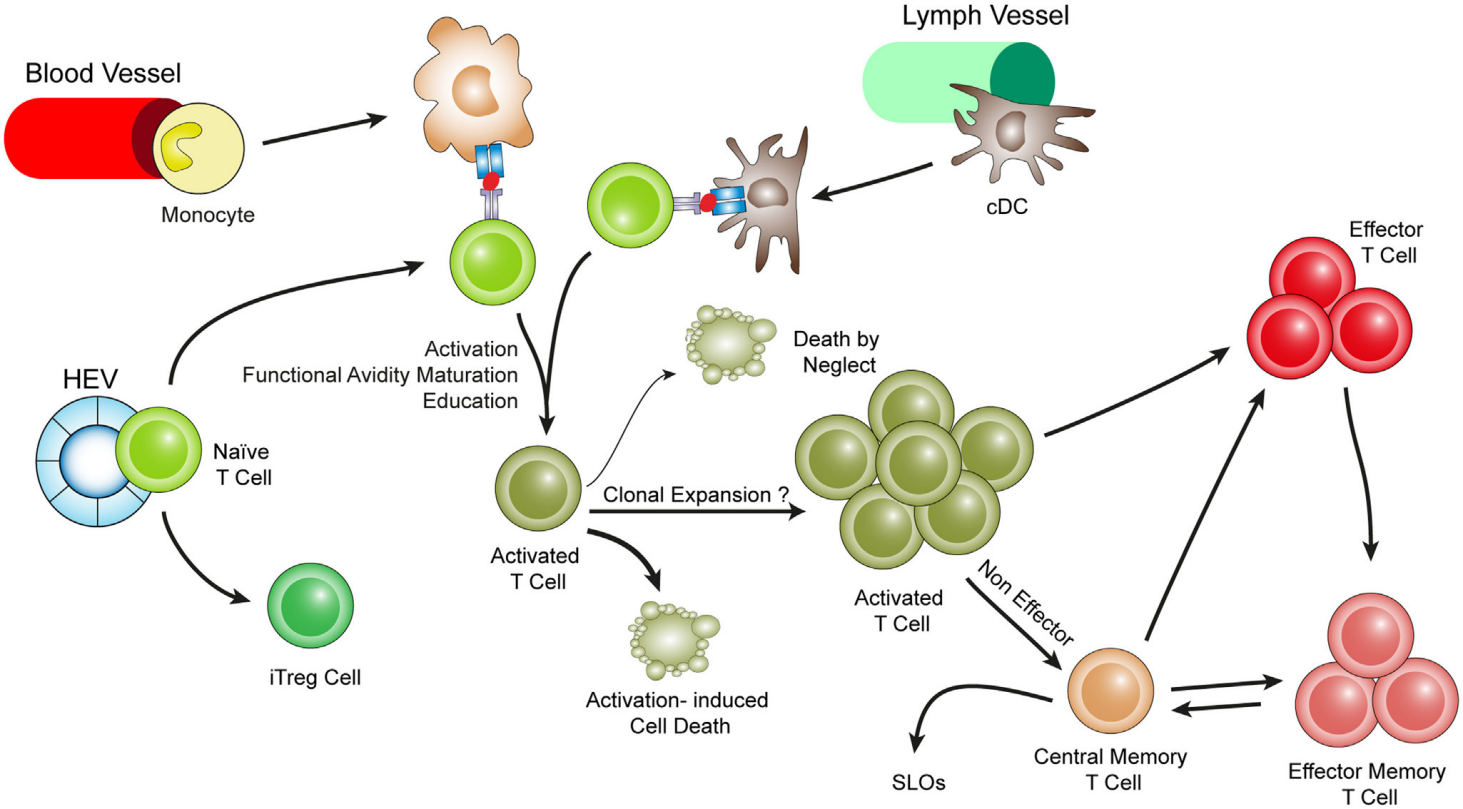
**Hypothetical choreography for antigen-dependent ATLO T-cell response pathways**. ATLOs are powerhouses of T-cell immunity. They promote T-cell recruitment and suppress T-cell egress allowing for extended T/DC interactions, activation, proliferation, and education of T-cells. ATLOs also mediate conversion of naïve CD4^+^ T-cells into iT_reg_ cells. This schematic representation has been modified from Hu et al. reported earlier [see Ref. (67) for detailed data].

**Figure 4:**
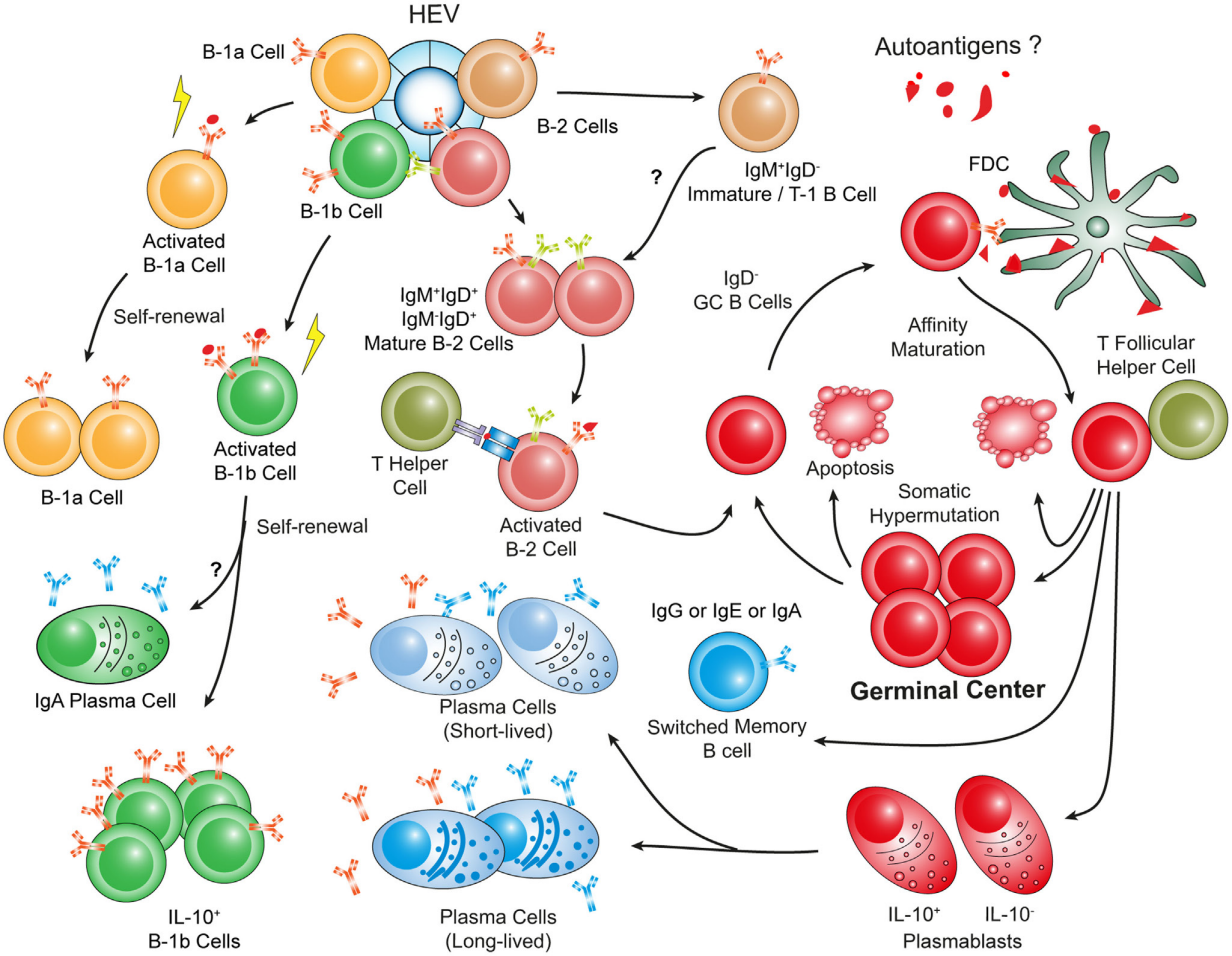
**Hypothetical choreography of antigen-dependent ATLO B-cell response pathways**. Advanced ATLOs are powerhouses of B-cell immunity. ATLO B-cell responses are two-pronged in nature containing a comprehensive B-1 and a multi-faceted B-2 maturation pathway. Although similarities of B-cell immunity in ATLOs and SLOs are apparent, differences include a large PC component and a distinct B-1 B cell compartment which differs from that in the peritoneal cavity. This schematic figure has been modified from Srikakulapu et al. (68) [see Ref. (68) for detailed data].

It should be noted, however, that ATLO stages and the onset of lymph vessel, blood vessel, and HEV neogenesis have not yet been defined. Following this reasoning, TLO Stage II can be distinguished from Stage I by the development of separate T- and B-cell areas. Although TLO Stage II lacking FDCs does not appear to be able to mount affinity maturation of B-cell receptors (BCRs) in a GC reaction [see Kranich and Krautler (51); this Research Topic], it is likely that activation of T-cells and B-cells and somatic hypermutation of BCRs can be organized in ATLOs stages I and II (67, 68). Finally, TLO Stage III shows activated GCs containing FDCs in B-cell follicles as their defining hallmark (see also Figures 3 and 4).

Although not directly demonstrated for any TLO, it is conceivable to assume that there is – at least for some TLOs – an antigen-driven T-cell activation and memory T-cell generation pathway (69, 70). In addition, in Stage III TLOs, an affinity maturation pathway leading to memory B-cells and/or PC generation is likely to occur (see below). Our proposal of TLO staging in general and that of ATLO staging in particular is debatable from a number of perspectives and should be regarded as an exercise to begin a discussion on the pros and cons of these definitions.

Our rationale to define the TLO stage is as follows: ATLOs Stage I already harbors proliferating T- and B-cells, which appear to be vigorously activated (note that both T-cell and B-cell activation occurs or can occur outside of GCs and in the absence of FDCs to mount a protective and/or proinflammatory adaptive immune response); the emergence of CCL21 and CXCL13 signals as strong drivers of lymphorganogenesis (71–74); CCL21 and CXCL13 expression indicate – in adult organisms – the activation of the lymphotoxin receptor and the tumor necrosis factor receptor on lymphoid tissue organizer (LTo) cells, which appear to be required for differentiation of stromal cells to become LTo cells (41, 67, 75–77).

It is of interest to note that major underlying mechanisms of ATLO neogenesis are distinct when compared with similar structures in the lung. Inducible bronchus-associated lymphoid tissues (iBALTs) preferentially form in young mice, whereas well-developed ATLOs are not observed before the age of 52 weeks (41, 78) [see article by Hwang et al. (79); this Research Topic]. However, there are also similarities, i.e., some iBALTs develop in the perivascular space of pulmonary blood vessels in addition to the connective tissue underneath the bronchial epithelium.

## ATLO Immune Cells and Structures

To understand atherosclerosis-related adaptive immune responses in the diseased arterial wall better, we determined leukocyte subtypes in the abdominal aorta during aging in ApoE^−/−^ mice, which we had tentatively termed *tertiary lymphoid follicles* (46, 66). As the high leukocyte density in these developing follicles prevented morphometric analyses, we established single cell suspension protocols of aorta segments to perform FACS analyses (67): T-cell subtypes include CD4^+^, CD8^+^, FoxP3^+^ CD4^+^, and few FoxP3^+^ CD8^+^ cells; a significant number of B-cells accumulate in the follicles though B-cells are rarely seen in other parts of the arterial tree; no lymphoid follicles develop in plaque-free aorta segments; leukocyte follicles are absent in young mice before 52 weeks of age; there is marked neogenesis of HEVs, lymph node-like conduits, and lymph vessels; the lymph vessels are aberrant in that they show dilated lumina with large numbers of luminal leukocytes; semiquantitative analyses revealed that the follicles promote recruitment of T-cells after adoptive transfer through HEVs, whereas little recruitment occurs in atherosclerotic plaques; the conduits resemble lymph node conduits as they exclude fluorescent dextran particles of 500 kDa (which were seen in the HEV lumen only) though they transport 10-kDa dextran particles; VSMCs in segments afflicted with atherosclerosis and lymphocyte follicles in the adventitia express lymphorganogenic chemokines CCL21 and CXCL13; *in vivo* blockade using an antagonistic decoy lymphotoxin β receptor antiserum eliminated FDCs from the aggregates and significantly reduced the number of HEVs (41). These data indicated that atherosclerosis is associated with TLO formation.

## Conduits, Angiogenesis, Lymphangiogenesis, and Neoformation of HEVs

There is considerable information on the structure and function of conduits in SLOs (41, 63, 80–83). In SLOs, stromal cells in the subcapsular sinus of lymph nodes play a major role in the traffic of molecules and of distinct immune cells (65, 84). Although little is known about conduits in TLOs, we identified conduit-like structures in ATLOs using immunohistochemistry and fluorescent dextran labeling *in vivo* [(41); Figure 2]. We observed that ATLO conduits share a series of structural and cellular similarities of LN conduits. Moreover, using fluorescently labeled microbeads, we observed that ATLO conduits sieve low molecular weight molecules from the circulation into the conduit network (41). However, the normal lymph node conduits are structures that connect afferent lymph and HEVs.

This anatomy of lymph node conduits is probably different from that of ATLOs: in lymph nodes, conduits form a reticular network connecting the subcapsular sinus and HEVs [see Ager (under review)^4^; this Research Topic]. Instead, we found that ATLO conduits connect the outer media of the arterial wall with newly formed HEVs within the T-cell areas of ATLOs. This distinctive anatomy of conduits in SLOs versus TLOs is interesting in view of the hypothesis that chemokine gradients may exist within ATLOs with possible CXCL13 derived from activated media segments adjacent to atherosclerotic plaques. Furthermore, small molecular weight soluble antigens could be transported from the media to T-cell areas or B-cell follicles. It is possible that the lymph vessels of ATLOs also connect with conduits, but this has not yet been studied. We therefore speculate that molecules in the range of the size of chemokines (which can enter conduits) and possibly soluble antigen may be transported from the media compartment to HEVs, i.e., the entry sites of lymphocytes into ATLOs. Given the capacity of several ATLO antigen-presenting cells (APCs) to present exogenous antigen to T-cells [see contribution of Maffia et al. (33); this Research Topic], ATLO conduits may therefore have functional roles in the maintenance of atherosclerosis immunity.

However, ATLOs lack capsules and therefore no apparent subcapsular sinus, i.e., the anatomical structure that allows afferent lymph vessels to drain cells and antigen *via* conduits into the parenchyma of the lymph node. Although there are clearly differences in conduit architecture between lymph nodes and ATLOs, the function of the latter may be to facilitate draining of the inflamed artery to allow antigen access the T-cell areas of ATLOs. However, the precise function of ATLO conduits remains to be determined. It will be of interest to test the possibility whether the atherosclerosis immune response within the arterial wall may be altered by crossing ApoE^−/−^ with PLVAP^−/−^ mice that have a pathological conduit system (82).

## Atherosclerosis, Aging, and ATLO T-Cell Immunity

Our data on ATLOs raise several questions: Is aging of the immune system (also referred to as immune senescence) (85–87) affected by hyperlipidemia? What is the relationship between local and systemic, i.e., SLO-dependent, atherosclerosis immunity during aging? Are there principal differences in T-cell immunity-related gene expression and inflammation-related gene expression in atherosclerotic plaques versus ATLOs? What is the territoriality of atherosclerosis T-cell responses in diseased arterial walls? (Figure 3) How are the ATLO T-cell responses organized as opposed to those in SLOs? Are VSMC lymphotoxin β receptors important in shaping the anatomy and function of ATLOs? Answers to these questions may be of general interest to understand TLO immunity in a wide range of disease conditions.

At the level of renal lymph nodes (RLNs) which drain the aorta, we characterized major T-cell subtypes during aging by FACS and genome-wide transcript expression arrays. Although the results of these studies identified major age-related gene expression changes, transcriptomes of WT versus ApoE^−/−^ RLNs were principally identical (67), indicating that hyperlipidemia does not affect T-cells and their activation systemically in major ways including aorta-draining LNs. However, transcriptome analyses of RLNs, spleen, and blood during aging showed marked aging/senescence-dependent changes. In sharp contrast, immune response-related and inflammation-related changes in aorta territories, i.e., plaques versus ATLOs, were dramatic, indicating that T-cell immunity in atherosclerosis appears to be regulated locally rather than systemically (67).

We used laser capture microdissection-based mRNA expression analyses to construct transcript atlases and to delineate the territoriality of T-cell immunity in the arterial wall in comparison with SLOs. Gene ontology (GO) terms *immune response, inflammation, T-cell activation, positive regulation of T-cell regulation*, and *T-cell proliferation* were examined. Again, these analyses failed to identify significant differential gene expression in WT versus ApoE^−/−^ RLNs (2 genes), but major changes between RLNs and ATLOs (1251 genes), between atherosclerotic plaques and ATLOs (1102 genes), and major changes between adventitia segments afflicted with versus those without atherosclerotic plaques in the intima (1274 genes). The transcript atlases of ATLOs and other affected diseased tissues in atherosclerosis (88, 89) will be important to address a variety of questions regarding atherosclerosis immunity and other issues including remodeling of extracellular matrix components during disease progression. The data were published in the NCBI Gene Expression Omnibus (GSE 40156) to be used to address a series of questions such as B-cell immunity in the arterial wall [see Ref. (68); below].

## T-Cell Phenotypes in ATLOs are Distinct from Those in SLOs

FACS analyses yielded additional information on the activation status of ATLO T-cell subtypes: naïve T-cells were rare in ATLOs yielding a 27-fold ratio of ATLO CD4^+^ T effector memory cells (T_EM_) and also considerable numbers of T central memory (T_CM_) T-cells over their naïve counterparts; similar data were obtained for T regulatory cells (T_reg_ cells) yielding an 87-fold ratio of T_reg_ cells with an EM or CM phenotype over their naïve counterparts, and in a similar, though less pronounced, way, the data were similar for CD8^+^ T-cells. Functional impacts of TLOs in general and of ATLOs in particular are only beginning to be understood. In peripheral tissues, immunosurveillance is carried out by tissue-specific homing and education of T_EM_ and T_CM_ T-cells as exemplified in skin and intestine (90–97). Thus, T-cells home into inflamed tissues, but they are also retained in these tissues to account for preferential action of T-cells in the diseased versus healthy tissues.

To study T-cell tropism in atherosclerosis, a series of adoptive transfer experiments were conducted to test the ability of ATLOs as *homing hubs* and *education centers* of T-cell immunity. Sizable numbers of ATLO CD4^+^, CD8^+^, and T_reg_ cells expressed CD103, PD-1, CD69, and other activation and homing molecules contrasting to their SLO counterparts. Moreover, there were little changes in these T-cell education signatures in WT versus ApoE^−/−^ SLOs. As T-cell recirculation is rapid, we used adoptive transfers using highly FACS-purified naïve T-cells in splenectomized and FTY720-treated mice: splenectomy was necessary to prevent recirculation of T-cells through the spleen, while FTY720 was required to prevent T-cell egress from lymph nodes. These data showed that ATLOs are effective in both recruitment and preventing egress of T-cells. FTY720 acts through sphingosine 1-phosphate receptors type 1 expressed by efferent lymph vessel endothelial cells in SLOs (98, 99). Our data also indicated that ATLO lymph vessels are functional and therefore may be involved in T-cell recruitment and recirculation into and out of the arterial wall bearing strong similarities to the function of lymph vessels in lymph nodes (67) (See text footnote 3).

Leukocyte movement in ATLOs versus WT adventitia was also assessed using multiphoton microscopy. These data showed striking similarities of T-cell movement that had been observed when naïve T-cells undergo a primary immune response in SLOs during activation and priming including length of migration per time interval, track velocity, and displacement (67, 100–102). In contrast, movement parameters of naïve T-cells in the WT adventitia showed nearly motionless cells. Detailed studies of movement parameters of T-cells in WT versus ApoE^−/−^ lymph nodes including the popliteal lymph node which does not drain the aorta showed identical characteristics. Furthermore, we identified a series of APCs in ATLOs using two independent assays of *in vivo* antigen presentation [see article of Maffia et al. (33); this Research Topic]. The composition of ATLO APCs, however, was aberrant in that the majority was mDCs followed by B-cells, cDCs, macrophages, and a minor population of lymphoid DCs (lyDCs).

## ATLOs Contain Large Numbers of T Regulatory Cells and Convert Naïve T-Cells into Induced T Regulatory Cells

T-cell-specific ATLO immunity is dichotomic in nature in that both proinflammatory and anti-inflammatory lymphocyte subsets including a large number of T_reg_ cells have been observed using immunohistochemistry and FACS analyses (41, 103). It is well established that T_reg_ cells accumulate in inflamed tissues (104–106). There are two major T_reg_ subtypes, i.e., one that is generated in the thymus (nT_reg_ cells) and another that is generated in the periphery from naïve CD4^+^ precursors (105) termed induced T_reg_ (iT_reg_) cells. The functional significance of these two T_reg_ subtypes for the regulation of T-cell immunity in various diseases remains to be fully understood, but it is possible that the TCR repertoire of naïve CD4^+^ T-cells gives rise to memory T_reg_ cells that have potent immunosuppressive properties under chronic inflammatory disease conditions as judged from other model systems in mice (105, 107). Moreover, regulatory T memory cells have been identified and may contribute to antigen-specific immunosuppression (108).

In view of our observation that ATLOs contain large numbers of T_reg_ cells (41), we attempted to characterize ATLO T_reg_ cells in more detail using subtype-specific markers and to delineate the activation status of ATLO T_reg_ cells. Naïve GFP-T_reg_ cells purified from spleens and lymph nodes of transgenic FoxP3-DTR-GFP mice (106) were adoptively transferred into aged ApoE^−/−^ mice and their phenotype was examined in ATLOs. The adoptively transferred T_reg_ cells did not show the activated endogenous phenotype even 3 weeks after transfer. These data were surprising and indicated that the endogenous ATLO T_reg_ cell population may be generated locally through an extended period of time during aging and that there may be clonal selection of endogenous T_reg_ cells in an antigen-specific way (67). We then examined the ability of ATLOs to convert CD4^+^ T-cells into iT_reg_ cells. FACS-purified naïve CD4^+^ T cells were transferred into aged ApoE^−/−^ mice. Although at 24 h, ATLOs did not show significant iT_reg_ cells, after 3 weeks, a considerable number of T_reg_ cells in ATLOs became Helios^−^/FoxP3^+^ iT_reg_ cells consistent with the ability of ATLOs to generate T_reg_ cells from CD4^+^/FoxP3^−^ precursors in the periphery (67).

## ATLO B-Cell Subsets Indicate Antigen-Specific Hypermutation, Affinity Maturation, and Isotype Switching in ATLO GCs

We explored aorta B-cell immunity in aged ApoE^−/−^ mice (Figure 4). Inspection of B-cell-related aorta transcriptomes (see above) revealed large numbers of differentially expressed transcripts in the GO terms *B-cell-mediated immunity, B-cell activation, positive regulation of B-cell-mediated immunit*y, *positive regulation of B-cell activation, B-cell proliferation*, and *B-cell differentiation* during aging (68). At the systemic immune system level, aging was associated with large numbers of age-dependent differentially expressed B-cell-related transcripts, but no major changes were observed in B-cell-related transcripts when WT and ApoE^−/−^ SLOs or blood were compared. These data demonstrated that the systemic B-cell transcriptome underwent strong aging/senescence-specific changes but that hyperlipidemia did not affect these changes.

B-cell-related transcripts of whole aortas specifically emerged during the time window of 52–78 weeks correlating with the appearance of ATLOs. *Bona fide* B-cell genes, such as IgM transcripts, were induced by a factor of up to 135. In sharp contrast, young WT or young ApoE^−/−^ aortas did not express B-cell genes as confirmed by immunohistochemistry. Furthermore, we never observed B-cells in atherosclerotic plaques either in the thoracic or abdominal aortas at any age using a variety of marker antibodies. However, as small lymph nodes lining the adventitia contained large numbers of B-cells, the FACS analyses – but not the immunohistochemical analyses – showed few B-cells indicating that the preparations of single cell suspensions from aortas were contaminated by lymph node-derived B-cells (109).

Laser capture microdissection-derived transcriptome atlases were constructed from distinct aorta tissues. Genes associated with B-cell survival, proliferation, differentiation, and activation were expressed in ATLOs including immunoglobulin genes, TACI (*tnfrsf13b*), B-cell activating factor receptor (*tnfrsf13c*), CD40 antigen (*cd40*), complement components (*c1qb*), and *myD88* (68). The degree of territoriality of adventitial B-cell transcripts was high as adventitial tissues adjacent to adventitia segments that were not afflicted with atherosclerotic lesions in the intima and did not or to a much lesser extent expressed these genes. Moreover, the *igj* chain gene involved in somatic hypermutation of the BCR, and B-cell memory cell generation (110) was expressed at a significant level in ATLOs. Expression of B-cell-related genes in the atherosclerotic plaque versus ATLOs showed marked differences: *bona fide* B-cell genes showed strong expression in the adventitia versus low expression in plaques [*ighm*; *cd19*; *ms4a1* (CD20), *igj*, and *cd79a/b*], whereas atherosclerotic plaques expressed inflammation-related B-cell-related genes that are expressed in B-cell target cells including macrophages. As expected from the global comparisons between WT and ApoE^−/−^ RLNs, no differential expression of B-cell-related genes were observed though – as pointed out above – aging/senescence was significant. We determined B-cell subtypes using an improved protocol to prepare single cell suspensions for FACS analyses (109).

## Heterogeneous B-Cell Subtypes in ATLOs Versus Lymph Nodes and Spleen

B-cell subtype data indicated three distinct major B-cell subtypes: B-2 cells, B-1 cells, and PCs. B-2 B-cell subtypes contained IgM^+^/IgD^−^ (immature or transitional B-cells are either immature B-cells that have left the bone marrow or they represent B-1 cells), IgM^+^/IgD^+^, and IgM^−^/IgD^+^ B-cells (mature B-cell maturation stages). Among mature IgD^+^ B-cells, IgM^−^/IgD^+^ B-cells represent follicular B-2 cells. IgM^−^/IgD^−^ cells represent either switched Ig^+^ B-cells, GC B-cells that have transiently lost Ig expression during somatic hypermutation of their Ig genes or they are GC memory B cells. Of special interest regarding impacts of ATLOs to control B-cell immunity was the identification of GC B-cells, i.e., IgD^−^/PNA^+^/GL-7^+^ B-2 cells, indicating GC reactions (somatic hypermutation and affinity maturation). These data indicated that ATLOs conduct the entire B-cell maturation pathway including antigen-specific B-cell GC reactions. Furthermore, we sought evidence for isotype switching within ATLOs, which follows the GC reaction in SLOs. We observed CD19^+^/IgD^−^/IgG1^+^, CD19^+^/IgD^−^/IgA^+^, and CD19^+^/IgD^−^/IgE^+^ B-2 cells in ATLOs. Although class switching is not restricted to GCs, the totality of our morphological, transcript atlas transcriptome, and FACS analyses are consistent with the possibility that class switching follows ATLO GC reactions (68).

## PCs Form a Large ATLO B-Cell Subset

We had observed earlier that ATLOs contain PCs in the periphery adjacent to T-cell zones (41). PCs constitute a major part of B-cell memory (29, 111, 112): two major PC subtypes had been identified in SLOs, i.e., the short-lived and long-lived PCs (113, 114), whereby short-lived PCs – after their generation – home to SLOs, whereas long-lived PCs largely home to the bone marrow. Both PC subtypes were identified in ATLOs using a BrdU labeling protocol (68). As it is known that PCs use inflammatory tissues as survival niches, our data fall short of demonstrating the generation of short-lived or long-lived PCs within ATLOs. Further work needs to address the important question where the ATLO PCs are generated and whether they provide B-cell memory that is atherosclerosis antigen-specific or whether they merely reflect the influx of atherosclerosis-unrelated PCs into the inflammatory arterial wall adventitia. In attempts to determine the potential functions of the arterial wall PCs, we determined their marker profile: a fraction of them express IL-10 indicating that at least some of them exert immunosuppressive activities. The second major B-cell subtype was B-1 cells. Two major observations deserve attention: (i) The B-1 cell subtypes, unlike their peritoneal cavity counterparts, were skewed toward B-1b cells versus B-1a cells. (ii) As the two B-1 subtypes have different functions to contribute to T-cell-independent B-cell immunity, further studies are needed to determine the functional impact of each of these subtypes to contribute to atherosclerosis immunity. As many of the ATLO B-1 cells express markers of immunosuppressive B regulatory cells (115–121) (TGFβ1, IL-10, PD-L1, and FasL), it is conceivable that the majority of B-1 cells have anti-atherosclerosis impacts on the disease.

## Exploration of TLOs: Tricks and Delusions Around Every Corner

As potential roles of TLOs in an expanding number of clinically important diseases are being recognized, robust methods for their exploration need to be established. ATLOs are among the best characterized TLOs in any disease: however, they are located in a complex tissue environment (see above) which asks for a combination of analytical techniques to avoid methodological flaws. Analyses of ATLOs – let alone other TLOs – pose considerable challenges: their formation occurs at inconsistent rates and variable sizes; ATLOs’ structures and cellular compositions range from small T/B lymphocyte aggregates to large lymphoid clusters containing distinct T-cell areas, B-cell follicles including activated GCs, and PC niches; similar to the dichotomic nature of SLOs, ATLOs generate both effector and immunosuppressive T and B lymphocytes; boundaries between ATLOs and other periaortic or perivascular tissues are difficult to define: they are located close to numerous small paraaortic lymph nodes, periaortic adipose tissue compartments, and ganglia of the sympathetic nervous system (Figure 1).

It is important to note: very little is known about these previously overlooked lymphoid aggregates (122). Consequently, major questions arose soon after they had been discovered: how to define a TLO? Should small T/B aggregates qualify as an early stage of a TLO or is the presence of FDCs in GCs essential to qualify a lymphocyte aggregate as TLO? How to distinguish between *bona fide* TLO cells and cells of neighboring tissues if tissue borders of the unencapsulated TLOs are ill-defined? Other challenges relate to determination of the size of TLOs, define the molecular cues of their interaction with the adjacent diseased tissue, determine their specific cellular constituents, quantify and determine the activation status of their immune cell subsets, characterize the properties and gene expression signatures of their connective tissue mesenchymal cells, and most importantly examine their functional impact on disease progression.

Analyses so far applied to ATLOs include laser capture microdissection-based large-scale microarray analyses. This method has been shown valuable to understand the territoriality of atherosclerosis-specific immune cells (41, 46, 67, 68, 88, 89, 109). If combined with immunohistochemical analyses and FACS analyses, it provides guidelines and specific information on many aspects of ATLO immunity. We have applied this technique to identify specific features of atherosclerosis immunity including lymphocyte activation and proliferation and the relation between artery immune responses and those in the draining RLNs. When carefully applied, laser capture microdissection-based mRNA expression analyses have helped to construct transcript atlases of both B-cell and T-cell immunity from which a series of conclusion can be drawn. These include lack of differential transcript expression in WT versus ApoE^−/−^ RLNs, a large inflammation-related transcriptome in ATLOs when compared to the RLN, lack of *bona fide* B-cell genes in plaques, and differences in gene expression in adventitia with atherosclerotic plaque versus adventitia without plaque. Another important technique applied to ATLOs has been FACS analyses together with morphometric assays to quantify distinct lymphocyte subsets and their activation status. However, when we performed comparative analyses of aorta single cell suspensions and immunofluorescent microscopy analyses, we became aware of distinct shortcomings of FACS analyses: since the adventitia or by the same token ATLOs are not visible with the naked eye, there was a tendency to detect immune cell subsets including B-cells in WT adventitia that were undetectable by immunohistochemistry indicating contamination of TLO preparations by neighboring tissues including FALCs.

## Are ATLOs Atherosclerosis Protective? Not so Fast!

In the past, TLOs have often been viewed as *disease-promoting* aggregates of immune cells largely because TLOs were exclusively *disease-associated* and because their size correlated with disease severity (123–126). However, as the immune cell composition of TLOs is being evaluated, concepts regarding their impact on disease have changed: it has been shown that immunosuppressive immune cells including T_reg_ cells, B-1 cells, and innate immune cells are major TLO constituents (Figures 1–4).

As mentioned above, we observed that VSMCs that are sandwiched between aorta atherosclerotic plaques and ATLOs express the lymphorganogenic chemokines, i.e., CCL21 and CXCL13. As these chemokines are both indispensable and sufficient to organize lymph nodes and TLOs [see contribution by Watanabe (127); this Research Topic], we hypothesized that VSMCs may adopt features of LTo cells. In cultured aorta VSMCs, we observed that a combination of TNF and agonistic antibodies directed against the lymphotoxin β receptor, but not each cytokine alone, led to a strong induction of CXCL13 mRNA and protein (77). To test the hypothesis that CXCL13 expression in VSMCs affects ATLO homeostasis, we generated ApoE^−/−^Ltbr^fl/fl^Tagln-cre mice using the late VSMC differentiation marker, i.e., Tagln/SM22α (67, 128). Unlike their Ltbr^−/−^ counterparts, ApoE^−/−^Ltbr^fl/fl^Tagln-cre mice showed no major alteration of the systemic immune system, indicating that the deletion of the Ltbr was specific for VSMCs. At the level of the arterial wall, however, ApoE^−/−^Ltbr^fl/fl^Tagln-cre mice showed two distinct phenotypes when compared to their ApoE^−/−^ controls: their ATLO structure was disrupted as evidenced by smaller ATLOs, less HEVs, loose mixed T-/B-cell aggregates rather than separate T- and B-cell areas; and the mice showed marked exacerbation of atherosclerosis. We interpreted these combined results as evidence that the VSMC lymphotoxin β receptor may be involved in atherosclerosis protection *via* ATLOs (67).

There are several caveats, however, regarding the veracity of this conclusion: VSMCs are also constituents of atherosclerotic plaques and the VSMC lymphotoxin β receptor may have affected atherosclerosis directly rather than *via* ATLOs; the mechanisms of an apparent atherosclerosis protection by ATLOs is complex as immunosuppressive leukocyte subsets have been described including T_reg_ cells (67) and a series of IL-10^+^ B regulatory cells (68). It is therefore possible that the promotion of atherosclerosis in ApoE^−/−^Ltbr^fl/fl^Tagln-cre mice is due to mechanisms other than *via* ATLO’s impact on the disease and that – under different conditions – ATLOs may even promote the disease. What these conditions may be will be a subject of future studies, but it could either include activation of the various effector lymphocyte subsets that have already been described to be major cellular constituents of ATLOs or the generation of autoimmune lymphocytes. Yet, we have shown – as a proof of principal experiment – that interference with the lymphotoxin β receptor in presumptive tissue-specific LTo cells is an experimentally feasible approach and that this interference has an effect on the outcome of the associated disease. Similar approaches could lead to interference with the lymphotoxin β receptor in lymphoid tissue-organizer-like cells in other disease models if the receptor can be targeted specifically as shown for the Tagln/SM22α gene.

## Challenges Ahead

We still do not know much about the specific impacts of any TLO on disease progression. Data from the characterization of the dichotomic nature of TLO immunity suggest that there is not a black or white picture: like SLOs, TLOs generate opposing immune responses that promote generation of antigen-specific lymphocyte subsets. Following this reasoning, it is probably naïve to give or ask for only one answer to this important question. However, we assume that the initial purpose of TLO neogenesis is to eliminate antigen and/or fight inflammation but whether and when the TLO turns into autoimmune responses and/or disease is – at present – difficult or impossible to assess. Analyses of the inflammatory cytokine/chemokine environment of TLOs and its chronicity versus the transient nature of antigen presentation in SLOs may yield additional distinguishing features of TLOs to examine their specific disease impacts.

Other distinguishing features of TLOs relate to the characterization of PC niches, the analysis of innate lymphoid cells, the composition and impact of the macrophage phenotypes in TLOs, and last but not least the T-cell receptor and BCR repertoire of TLOs versus those of SLOs. One approach to identify the specific impact of TLOs in disease may be to delete the lymphotoxin β receptor in presumptive LTo cells as exemplified for ATLOs in atherosclerosis.

Although it seems clear from TLO research during recent years that disease-associated lymphoid clusters are powerhouses of disease immunity, new experimental approaches are needed. These include next-generation sequencing, specific tissue-targeted disruption of the lymphotoxin β receptor or of lymphorganogenic chemokines, improved methods to identify potential antigen-specific lymphocytes, a better understanding and characterization of iT_reg_ cells, isolation of B memory cells and PCs, and cloning of their immunoglobulin genes. It will be of utmost interest to study tolerance mechanisms in TLOs. If TLOs fail to maintain tolerance, autoimmune lymphocytes are likely to be generated in them, which may have major implications for disease immunity. Understanding TLO immunity better may lead the way to exploit the amazing ability of the immune system to target disease mechanisms by developing immunotherapeutics for both chronic autoinflammatory and autoimmune diseases.

## Conclusion

Artery tertiary lymphoid organs are complex atherosclerosis-associated lymphoid aggregates that generate dichotomically acting immunosuppressive and immune response-promoting immune cells. Their structural organization and cellular composition suggest three major pillars of disease immunity: a large inflammatory component, a considerable antigen-independent element, and a possible antigen-specific domain. ATLO structures promote immune responses by recruitment of naïve lymphocytes, impaired egress, and extended DC/lymphocyte cluster formation. Until today, although many TLOs occur in autoimmune diseases, TLOs have not been demonstrated to generate autoreactive lymphocytes.

## Author Contributions

All authors contributed to the design, writing, and editing of the submitted manuscript.

## Conflict of Interest Statement

The authors declare that the research was conducted in the absence of any commercial or financial relationships that could be construed as a potential conflict of interest.

## Footnotes

^1^Hughes C, Bedaj M, Benson RA, Maffia P. Antigen presenting cells and antigen presentation in tertiary lymphoid organs. (under review in this Research topic).

^2^Caamano J et al. (manuscript in preparation for this Research topic).

^3^Ruddle N. High endothelial venules and lymphatic vessels in tertiary lymphoid organs: characteristics, functions, and regulation. (under review in this Research topic).

^4^Ager A. High endothelial venules. (manuscript in preparation for this Research topic).
